# Septoplasty Versus Non-Surgical Treatment for Deviated Nasal Septum: A Systematic Review and Meta-Analysis

**DOI:** 10.3390/jcm15103930

**Published:** 2026-05-20

**Authors:** Uday Abd Elhadi, Alaa Safia, Muhamed Masalha

**Affiliations:** 1 Department of General Medicine, Ziv Medical Center, Safed 24908, Israel; 2 Department of Otolaryngology, Ziv Medical Center, Safed 24908, Israel; 3 Department of Otolaryngology, Emek Medical Center, Afula 1834111, Israel

**Keywords:** deviated nasal septum, septoplasty, non-surgical treatment, meta-analysis

## Abstract

A deviated nasal septum (DNS) is a frequently observed anatomical abnormality that can lead to nasal airflow obstruction and reduced quality of life. Although septoplasty is widely regarded as the standard surgical approach for symptomatic cases, its effectiveness compared with conservative, nonsurgical treatments remains a subject of ongoing debate. **Methods**: We performed a systematic literature review up to 26 December 2025, using databases including PubMed, EMBASE, CENTRAL, and Web of Science. The review included only randomized controlled trials (RCTs) involving adult patients with DNS that compared septoplasty to non-surgical treatment strategies. **Results**: A total of three studies were included in the final analysis. Septoplasty significantly improved NOSE scores at 3 months (MD −12.58, 95% CI −19.10 to −6.06), 6 months (MD −28.73, 95% CI −33.44 to −24.03), and 12 months (MD −17.27, 95% CI −22.85 to −11.69) compared to non-surgical management. Similarly, SNOT-22 scores favored septoplasty at all time points, with the largest benefit observed at 6 months (MD −9.64, 95% CI −12.66 to −6.62). PNIF scores showed improvement in the septoplasty group at 6 months (MD 15.26, 95% CI 4.20 to 26.31), though significance varied over time. **Conclusions**: In adults with deviated nasal septum, the currently available randomized evidence suggests that septoplasty may provide greater improvement in symptoms and quality of life compared with non-surgical approaches. However, these findings should be interpreted cautiously due to the limited number of included studies and the low-to-moderate certainty of evidence.

## 1. Introduction

Deviated nasal septum (DNS) is a common anatomical condition, reported in up to 80% of the general population, though only a subset of patients present with clinically significant symptoms requiring intervention [1,2]. DNS occurs when the nasal septum, the cartilage and bone separating the nasal cavities, is displaced from the midline, leading to impaired airflow and nasal obstruction. Symptoms associated with DNS include nasal congestion, difficulty breathing through the nose, snoring, facial pressure, sleep disturbances, and recurrent sinus infections, all of which may significantly impair quality of life [3,4].

DNS can be managed through conservative, medical, and surgical methods. Non-surgical treatments, including intranasal corticosteroids, antihistamines, and decongestants, focus on reducing mucosal inflammation and alleviating symptoms but do not address the underlying structural deformity [5]. Septoplasty, a surgical intervention aimed at straightening the DNS, is the definitive solution for symptomatic DNS that does not respond to medical therapy. It ranks among the most commonly performed procedures in the field of otolaryngology [6,7].

Although septoplasty is commonly performed, its effectiveness compared with non-surgical treatments remains a topic of debate, especially for patients with mild-to-moderate symptoms. Some studies indicate significant improvements in nasal airflow and overall quality of life after septoplasty [8,9]. Other studies point to a considerable placebo effect and suggest minimal advantages for specific groups [10]. Variations in study design, outcome measures, and patient selection criteria complicate the ability to reach clear conclusions.

This study aims to systematically review and analyze evidence from randomized controlled trials (RCTs) that compare septoplasty to non-surgical treatments in individuals with DNS. The aim is to evaluate and compare their effectiveness in alleviating symptoms, enhancing quality of life, improving patient satisfaction, and minimizing adverse events. By integrating the most reliable data, this review seeks to offer clearer, evidence-based recommendations to support clinical decision-making in the management of DNS.

## 2. Materials and Methods

### 2.1. Protocol Registration

**Figure 1:**
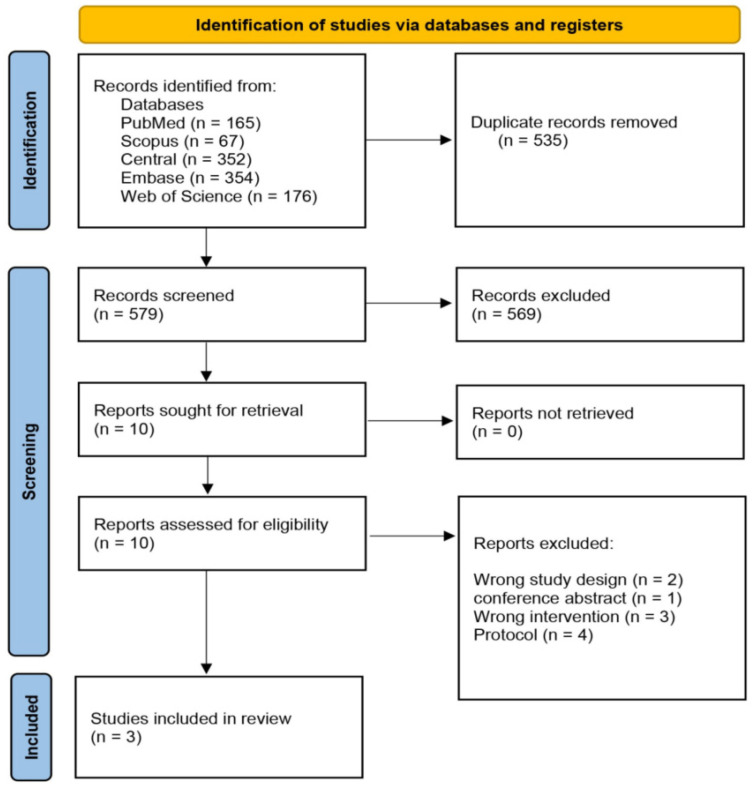
The PRISMA (Preferred Reporting Items for Systematic Reviews and Meta-Analyses) guidelines and was aligned with the methodological principles recommended in the Cochrane Handbook for Systematic Reviews.

This systematic review and meta-analysis was conducted according to the Preferred Reporting Items for Systematic Reviews and Meta-Analyses (PRISMA 2020) guidelines. The study protocol was previously registered on PROSPERO (CRD420261347686). Approval from the Institutional Review Board was waived due to the non-involvement of human participants in this research. (Figure 1 and Table S2).

### 2.2. Databases and Search Strategy

A comprehensive literature search was conducted through 26 December 2025, across the following databases: PubMed (MEDLINE), Web of Science (WoS), EMBASE, and the Cochrane Central Register of Controlled Trials (CENTRAL). Tailored search strategies and keywords were applied for each database to maximize sensitivity and relevance. In addition, a manual screening of reference lists was performed to identify any additional eligible studies not captured during the electronic search (Table S1).

### 2.3. Screening and Selection Criteria

This study encompassed research aligned with the following PICO criteria:

Population (P): Adults aged 18 and above experiencing nasal obstruction due to a deviated nasal septum (DNS), confirmed through clinical examination and imaging.

Intervention (I): Septoplasty, with or without turbinate reduction.

Comparison (C): Non-surgical approaches such as intranasal corticosteroids, saline irrigation (including isotonic solutions), nasal decongestants, or observation.

Outcomes (O): Efficacy assessed by validated tools like the Nasal Obstruction Symptom Evaluation (NOSE), Sino-Nasal Outcome Test (SNOT-22), and Peak Nasal Inspiratory Flow (PNIF). Safety outcomes included rates of postoperative complications such as bleeding, infection, septal perforation, and revision procedures. The primary endpoint was improvement in quality of life.

Study Design (S): Only RCTs published in peer-reviewed journals were considered.

### 2.4. Study Selection

The review utilized the Covidence online platform. After removing duplicates, four authors independently assessed each record. They examined the full texts during the initial screening to determine eligibility criteria. Any disagreements were resolved through discussions.

### 2.5. Data Extraction

To accurately establish the data extraction sheet, we conducted a pilot extraction after securing the full texts of the relevant publications. The Excel (Microsoft, Redmond, DC, USA) data extraction sheet is organized into three sections: (1) a summary of included studies, detailing the name of the first author, year of publication, study design, country, recruitment duration, sample size, study arms, and follow-up duration; (2) a baseline characteristics sheet containing participant details such as sample size, age, sex, nasal obstruction, septal deviation, and smoking status; (3) outcomes data including efficacy, safety, and quality of life.

### 2.6. Risk of Bias and Certainty of Evidence

Two reviewers independently evaluated the methodological quality of the included randomized controlled trials (RCTs) using the Cochrane Risk of Bias 2 (ROB 2) tool (Cochrane Collaboration, https://methods.cochrane.org/risk-bias-2 (accessed on 10 May 2026)). This tool focuses on five critical areas: the randomization process, deviations from planned interventions, missing outcome data, measurement of outcomes, and selection of reported results. Studies were categorized based on these areas as having “low risk of bias,” “some concerns,” or “high risk of bias.” Any disagreements between the reviewers were resolved through discussions with a third reviewer. Furthermore, potential publication bias was taken into account. To determine the certainty of the evidence for each outcome, we applied the Grading of Recommendations Assessment, Development and Evaluation (GRADE) approach, which assesses various factors, including risk of bias, inconsistency, indirectness, imprecision, and other pertinent considerations. The overall quality of the evidence was then rated as high, moderate, low, or very low.

Although all included studies were assessed as low risk of bias using the ROB 2 tool, most trials were open-label in design. The lack of blinding may introduce performance and detection bias, particularly for subjective outcomes such as NOSE and SNOT-22 scores, which should be considered when interpreting the results.

### 2.7. Statistical Analysis and Trial Sequential Analysis

All statistical analyses were conducted using RevMan version 5.4. For continuous efficacy outcomes, mean differences (MD) with 95% confidence intervals (CI) were calculated. A random-effects model was applied throughout to account for potential variability among studies. Heterogeneity was assessed using the Chi-square test and the I^2^ statistic; I^2^ values above 50% indicate substantial heterogeneity.

A Trial Sequential Analysis (TSA) was conducted to assess the strength, stability, and reliability of the pooled evidence from the included randomized controlled trials. TSA is a statistical approach that combines elements of cumulative meta-analysis with sequential monitoring boundaries, enabling researchers to determine whether the available evidence is sufficiently conclusive or warrants further studies. By calculating the Required Information Size (RIS), the sample size needed to achieve adequate statistical power, TSA helps identify the point at which the cumulative data become strong enough to confirm a genuine treatment effect. This method minimizes the likelihood of random errors and mitigates the risks of both Type I (false-positive) and Type II (false-negative) findings that may occur when meta-analyses are repeatedly updated as new trials emerge.

Importantly, TSA demonstrated that while short-term outcomes (3 months) may still be underpowered and subject to random error, the evidence for mid-term (6 months) and long-term (12 months) outcomes appears robust and conclusive. This distinction highlights the need for cautious interpretation of early outcomes while supporting the reliability of sustained benefits.

## 3. Results

### 3.1. Characteristics of Included Studies

The baseline details and characteristics of three RCTs with 721 patients included are mentioned in Table 1 and Table 2.

The non-surgical comparison arms varied among the included trials and consisted of different combinations of intranasal corticosteroids, saline irrigation, decongestants, standard medical therapy, or observation protocols.

### 3.2. Risk of Bias

**Figure 2:**
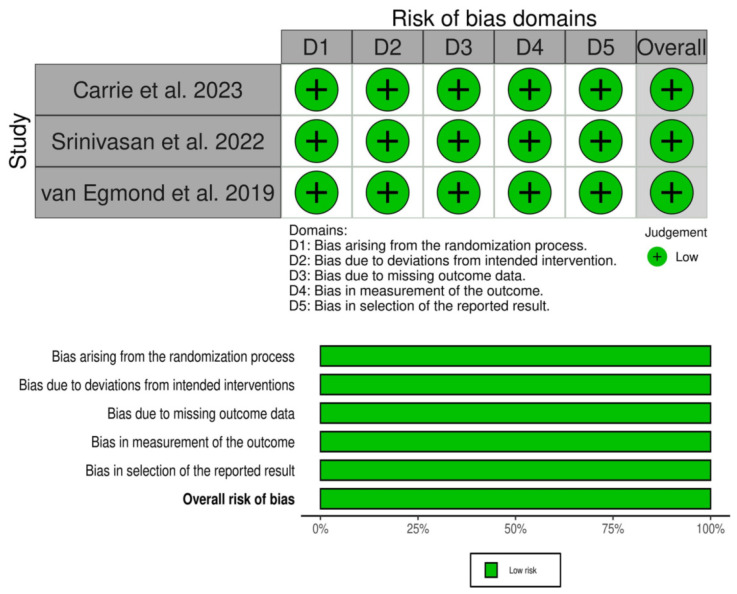
Risk of bias assessment of included randomized controlled trials using the Cochrane Risk of Bias 2 (RoB 2) tool. In-cluded studies: Carrie et al., 2023 [11]; Srinivasan et al., 2022 [12]; and van Egmond et al. [13], 2019. D1: bias arising from the randomization process; D2: bias due to deviations from intended interventions; D3: bias due to missing outcome data; D4: bias in measurement of the outcome; D5: bias in selection of the reported result.

All studies included showed low risk of bias; details of ROB2 and GRADES are presented in Figure 2 and Table 3.

### 3.3. Meta-Analysis

#### 3.3.1. Primary Outcomes

**Figure 3:**
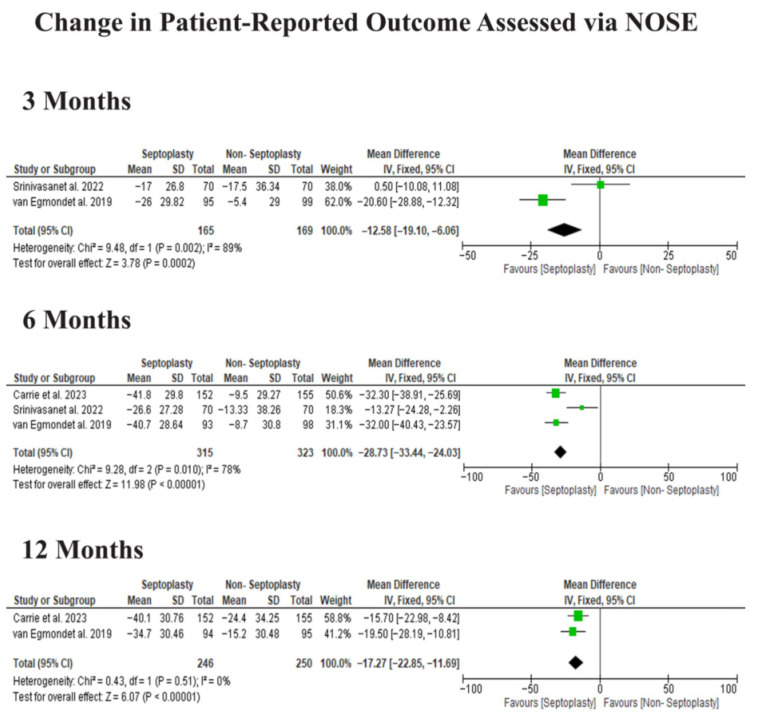
Change in NOSE scores across follow-up periods after septoplasty versus non-surgical treatment. Data derived from Carrie et al. [11], Srinivasan et al. [12], and van Egmond et al. [13].

Across all time points, patients who underwent septoplasty showed significantly greater improvements in NOSE scores compared to those treated non-surgically. At 3 months, the MD favored septoplasty (MD −12.58, 95% CI −19.10 to −6.06, *p* = 0.0002). The difference was more pronounced at 6 months (MD −28.73, 95% CI −33.44 to −24.03, *p* < 0.00001; I^2^ = 78%). At 12 months, the improvement remained statistically significant (MD −17.27, 95% CI −22.85 to −11.69, *p* < 0.00001), Figure 3.

#### 3.3.2. Secondary Efficacy Outcomes

##### Patient-Reported Outcome Assessed via SNOT-22 Scale

**Figure 4:**
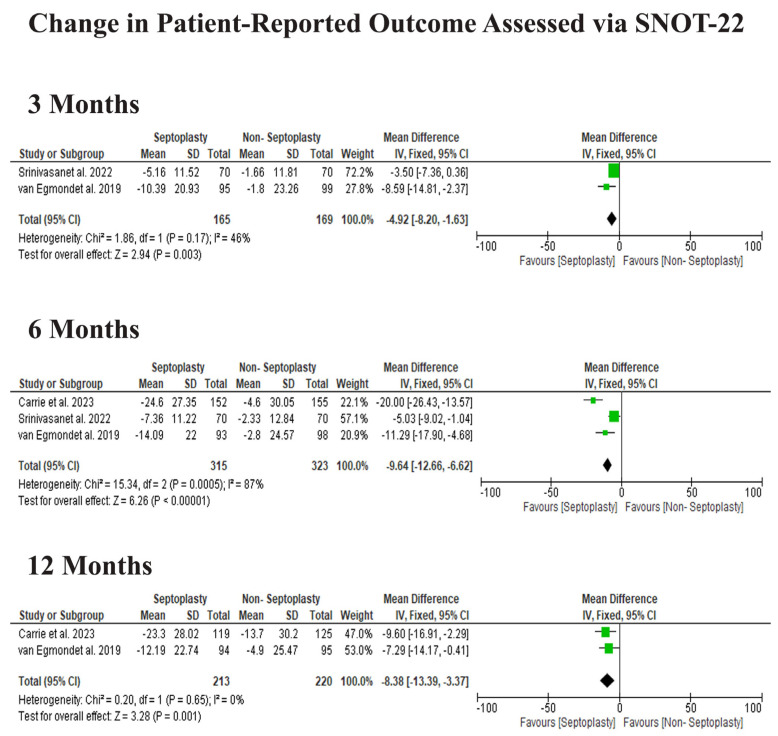
Change in SNOT-22 scores across follow-up periods after septoplasty versus non-surgical treatment. Data derived from Carrie et al. [11], Srinivasan et al. [12], and van Egmond et al. [13].

Patients undergoing septoplasty demonstrated significantly greater improvement in SNOT-22 scores compared to those treated non-surgically. At three months, the mean difference (MD) favored septoplasty (MD −4.92, 95% CI −8.20 to −1.63, *p* = 0.003). This effect was even more pronounced at six months (MD −9.64, 95% CI −12.66 to −6.62, *p* < 0.00001). Over 12 months, the benefit remained statistically significant (MD −8.38, 95% CI −13.39 to −3.37, *p* = 0.001; Figure 4).

#### 3.3.3. Patient-Reported Outcome Assessed via PNIF

**Figure 5:**
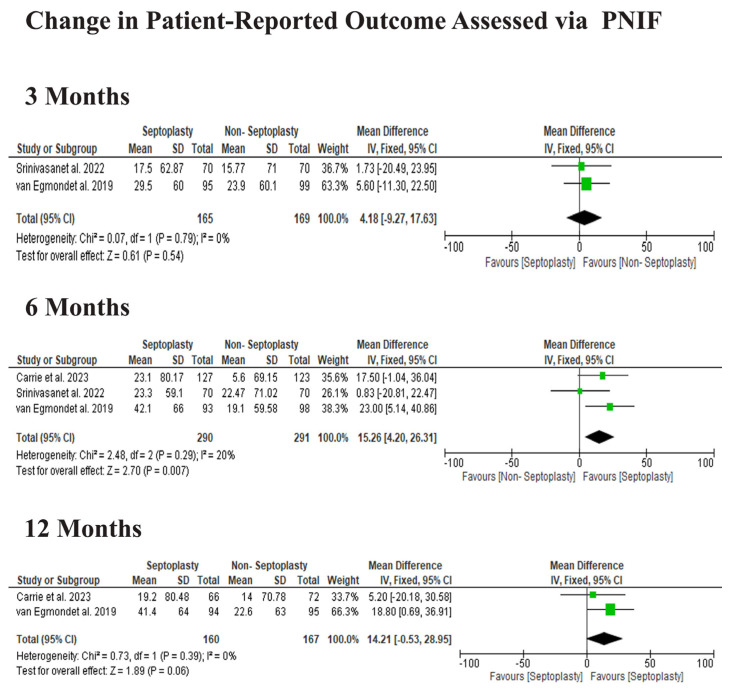
Change in PNIF scores across follow-up periods after septoplasty versus non-surgical treatment. Data derived from Carrie et al. [11], Srinivasan et al. [12], and van Egmond et al. [13].

Septoplasty was associated with improved PNIF scores compared to non-surgical management, though the statistical significance varied across time points. At 3 months, the MD favored septoplasty but did not reach statistical significance (MD 4.18, 95% CI −9.27 to 17.63, *p* = 0.54). At 6 months, a significant improvement in PNIF was observed in the septoplasty group (MD 15.26, 95% CI 4.20 to 26.31, *p* = 0.007). By 12 months, the difference remained in favor of septoplasty but was not statistically significant (MD 14.21, 95% CI −0.53 to 28.95, *p* = 0.06), Figure 5.

#### 3.3.4. Safety Outcomes

**Figure 6:**
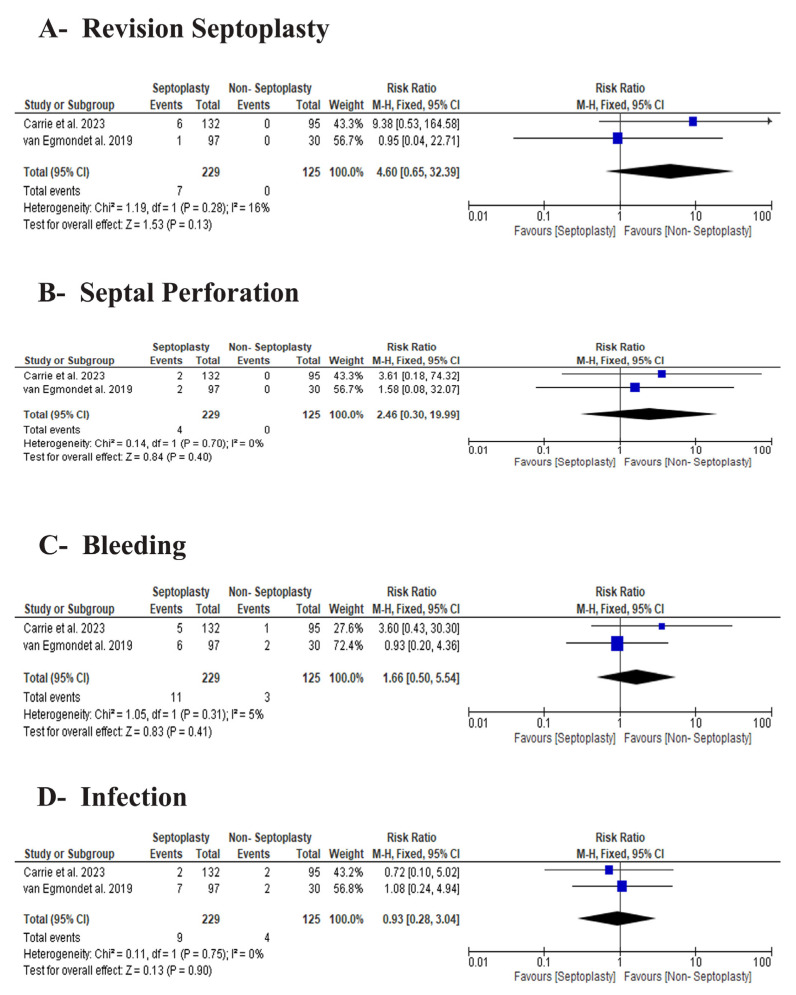
Safety outcomes comparing septoplasty and non-surgical treatment. Data derived from Carrie et al. [11], Sriniva-san et al. [12], and van Egmond et al. [13].

There were no significant differences in revision septoplasty between the two groups (RR 4.60, 95% CI 0.65 to 32.39, *p* = 0.13), the rates of septal perforation (RR 2.46, 95% CI 0.30 to 19.99, *p* = 0.40; I^2^ = 0%), bleeding (RR 1.66, 95% CI 0.50 to 5.54, *p* = 0.41; I^2^ = 0%), and infection (RR 0.93, 95% CI 0.28 to 3.04, *p* = 0.90; I^2^ = 0%), Figure 6.

Trial Sequential Analysis (TSA) for Primary Outcomes.

At 3 months, the TSA estimated a Required Information Size (RIS) of 1672 participants, whereas the cumulative sample size in the meta-analysis was 334. The cumulative Z-curve crossed the conventional significance boundary (Z = ±1.96), indicating that septoplasty was statistically superior to non-surgical management in reducing nasal obstruction symptoms in the short term. However, the Z-curve did not cross the trial sequential monitoring boundary nor reach the RIS threshold. This suggests that, while the observed effect is statistically significant, it may still be prone to random error due to insufficient accumulated information. Therefore, the short-term benefit of septoplasty should be interpreted with some caution, and additional well-powered RCTs are needed to validate this early improvement (Figure S1).

At 6 months, the RIS was calculated to be 236 participants, with a cumulative sample size of 191. The cumulative Z-curve crossed both the conventional significance boundary and the trial sequential monitoring boundary before reaching the RIS line. This pattern indicates that the available evidence has already exceeded the statistical threshold for confirming a firm and conclusive effect of septoplasty over non-surgical management at 6 months. The crossing of both boundaries signifies that the cumulative evidence is sufficiently robust to rule out type I error, even with a slightly smaller sample size than the theoretical requirement. Thus, TSA confirms that septoplasty produces clinically and statistically reliable improvement in nasal obstruction and quality of life at the intermediate follow-up period (Figure S2).

For the NOSE outcomes at 12-month follow-up, the RIS was 197 participants, with an accumulated sample size of 189. Similar to the 6-month analysis, the Z-curve crossed both the conventional and the trial sequential monitoring boundaries before reaching the RIS. This demonstrates that the effect estimate is both statistically significant and conclusively reliable, indicating that further trials are unlikely to overturn this finding. The sustained crossing of both boundaries supports the long-term durability of septoplasty’s therapeutic benefit, showing persistent improvement in nasal airflow and symptom relief up to one year post-surgery (Figure S3).

## 4. Discussion

In this meta-analysis of three RCTs, septoplasty was compared with non-surgical management in adult patients with symptomatic DNS and was found to improve objective and patient-reported outcomes more consistently and sustain those improvements. Reductions in NOSE and SNOT-22 scores, indicative of nasal obstruction severity, along with sinonasal quality of life, were observed post-intervention at 3, 6, and 12 months, respectively, and were significant and substantial. A prominent increase was also demonstrated at 6 months through PNIF. Safety profiles were comparable between surgical and conservative approaches. There were no statistically significant differences in bleeding rates, infection rates, septal perforation rates, or the need for revision surgery. The effectiveness of septoplasty is well documented by substantial improvements in NOSE scores (MD −28.73 at 6 months) and SNOT-22 scores (MD −9.64 at 6 months). This surgical procedure mitigates both quality-of-life and functional burdens associated with DNS. The gains from PNIF are significant only after 6 months and are temporary, likely due to gradual remodeling that occurs after the initial mucosal recovery and the decrease in edema after surgery. These results align with typical outcomes after septal correction: the immediate mechanical realignment quickly relieves symptoms, while individual healing patterns help maintain the correction over the long term.

The total estimates from the study reinforce the conclusions of prior cohort studies and single-center RCTs reporting superior symptomatic relief following septoplasty compared with medical therapy (e.g., intranasal corticosteroids or saline irrigation) [4,14]. For instance, a study reported a mean NOSE reduction of 25 points at 6 months post-septoplasty, whereas non-surgical arms showed minimal change. Similarly, Chang and colleagues, in a 2022 survey of 202 patients, observed significant improvements in SNOT-22 scores following septoplasty, consistent with our results [15]. However, unlike observational studies that sometimes report higher complication rates, our analysis of RCT data suggests no increased surgical risk under control, standardized protocols [12].

The findings of our meta-analysis, which demonstrate that septoplasty provides significantly greater symptom relief and quality-of-life improvements compared to non-surgical treatment in adults with DNS, are consistent with the growing body of randomized evidence in recent research. For example, A study conducted a pragmatic RCT in the Netherlands and found that 78% of patients undergoing septoplasty achieved clinically significant improvements in NOSE scores at 24 months, compared to only 46% in the non-surgical group, underscoring the long-term benefit of surgical correction [13].

Similarly, the UK-based trial researchers found that septoplasty led to a 20-point greater reduction in SNOT-22 scores at 6 months versus medical management alone, far exceeding the minimal clinically significant difference, with consistent improvements in PNIF and SF-36 scores [11]. Our study also mirrors the results of a 2022 survey, which showed that septoplasty produced significantly greater improvements in nasal obstruction VAS, NOSE, and SNOT-22 scores than conservative therapy, with gains maintained over 6 months [12]. These outcomes align with a meta-analysis by another study, which pooled three RCTs and confirmed that septoplasty significantly improved NOSE and SNOT-22 scores at 6 and 12 months, though not at 3 months, matching the temporal pattern observed in our pooled analysis [16].

Wu et al. (2024) found substantial benefits from septoplasty in patients with DNS and allergic rhinitis, reporting considerable standardized improvements in nasal obstruction and total nasal symptom scores, suggesting that surgical correction enhances medical therapy in such comorbid patients [17]. Another study compared outcomes in DNS patients with and without septoplasty and found significantly greater improvements in nasal airflow and patient-reported symptoms in the surgical group, even in cases of mild deviation [18]. Likewise, this study [19] demonstrated that septoplasty improved PNIF by 15–20 L/min, a magnitude comparable to the 15.26 L/min improvement reported in our study at 6 months.

In terms of safety, our findings are corroborated by these two studies, which reported low rates of adverse events such as bleeding or infection (<5%), and minimal risk of septal perforation or revision surgery [13,16]. Moreover, a trial in a study showed that adding turbinate reduction to septoplasty offered no additional benefit in mild-to-moderate DNS cases, supporting the generalizability of our results, which pooled data from studies with or without turbinate surgery [20]. Finally, a recent RCT reported that 89% of patients undergoing septoplasty would recommend the procedure to others, emphasizing patient satisfaction and reinforcing the superior quality-of-life outcomes observed in our pooled NOSE and SNOT-22 analysis [6]. Collectively, these findings validate our conclusion that septoplasty offers significantly greater efficacy than conservative therapy across multiple validated endpoints and diverse patient populations.

### 4.1. Strengths and Limitations

The strengths of this meta-analysis include adherence to PRISMA and Cochrane methodologies, comprehensive database coverage, a focused focus on RCTs, and a meticulous assessment of risk of bias. The implementation of validated outcome measures across multiple time points further augments confidence in the temporal trajectory of benefits. However, the limitations of this study are highlighted by the small number of available RCTs, which restricts statistical power for specific subgroups and safety analyses.

The moderate-to-high heterogeneity observed in some outcomes (e.g., NOSE score at 6 months, I^2^ = 78%) may be attributed to several factors, including variations in surgical techniques (endoscopic vs conventional septoplasty), differences in the use of concurrent turbinate reduction, heterogeneity in non-surgical management strategies (such as intranasal corticosteroids, saline irrigation, or observation), and differences in baseline patient characteristics and symptom severity.

A key limitation of this study is the small number of included randomized controlled trials (n = 3), which may limit the statistical power and generalizability of the findings. Although Trial Sequential Analysis provided additional support for the robustness of mid- and long-term outcomes, the overall certainty of evidence remains low to moderate according to GRADE assessment. Therefore, the results should be interpreted with caution.

Another important limitation is the heterogeneity of non-surgical treatment protocols across studies. These included varying combinations of intranasal corticosteroids, saline irrigation, decongestants, and observation. Such variability may influence treatment outcomes and reduce the precision of comparisons between surgical and non-surgical approaches.

Additionally, the comparison arms differed across the three included randomized controlled trials. While all studies compared septoplasty with conservative management, the specific non-surgical strategies varied and included combinations of intranasal corticosteroids, saline irrigation, topical decongestants, watchful waiting, and standard medical therapy. Furthermore, some studies included concurrent turbinate reduction in selected surgical patients, whereas others evaluated isolated septoplasty. These differences in treatment protocols may partially explain the heterogeneity observed in several pooled outcomes, particularly NOSE scores at 6 months. Nevertheless, despite these variations, the direction of effect consistently favored septoplasty across all included studies and major patient-reported outcomes, supporting the overall robustness of the findings.

However, this heterogeneity also reflects real-world clinical practice, where non-surgical management is not standardized and is often tailored to individual patient characteristics.

### 4.2. Future Implications

This meta-analysis reveals that septoplasty is more clinically effective than conservative treatments in enhancing disease-specific quality of life and alleviating nasal obstruction in adults with DNS.

Firstly, septoplasty provides clear symptomatic relief, but the long-term durability of its benefits, especially beyond the one-year follow-up period, remains insufficiently studied. Most included RCTs, including the NAIROS trial [18], assessed outcomes only up to 6 or 12 months, leaving uncertainty about symptom recurrence, sustained airflow improvement, and late complications such as septal re-deviation or scarring. Future trials should include extended follow-up (2–5 years) to assess the stability of patient-reported outcomes and objective measures such as PNIF.

Secondly, there is growing interest in personalized treatment algorithms for DNS. Although septoplasty is effective on average, it may not benefit all patients equally. Factors such as concomitant allergic rhinitis, turbinate hypertrophy, or psychological comorbidities (e.g., anxiety-related symptom perception) can modify response to surgery [21]. Future research should aim to stratify outcomes by patient phenotype, using baseline NOSE scores, nasal airflow resistance, mucosal inflammation, and quality-of-life indices to guide individualized management. Incorporating biomarkers (such as eosinophilic inflammation) along with nasal endoscopy grading can enhance patient care selection.

Thirdly, cost-effectiveness remains a key issue in public health planning. Therefore, longitudinal economic analyses in different health systems, especially in low- and middle-income countries, are needed to inform resource allocation. Additionally, it is essential to create shared decision-making tools to assist clinicians and patients in selecting between surgical options and ongoing medical treatment. Research indicates that decision aids increase patient satisfaction and compliance, while also minimizing regret associated with elective surgeries [22]. In conclusion, upcoming RCTs should examine the effects of adjunctive or staged interventions, such as concurrent inferior turbinate reduction, as these may improve surgical outcomes for patients with both structural and mucosal conditions. Additionally, research comparing endoscopic and open septoplasty methods, along with the influence of surgeon expertise and hospital volume on outcomes, will enhance procedural standardization and improve safety.

It is also important to recognize that short-term response to nasal decongestants may serve as a predictor of surgical outcomes in some patients. However, decongestants provide only temporary symptomatic relief and do not correct the underlying structural deviation. In contrast, septoplasty addresses the anatomical cause of obstruction and provides more sustained improvement over time, as supported by our pooled analysis and TSA findings.

Despite the limited number of available randomized controlled trials, this review provides an updated synthesis of the highest level of comparative evidence currently available on septoplasty versus conservative management for deviated nasal septum. By integrating patient-reported outcomes, objective airflow measures, GRADE certainty assessment, and Trial Sequential Analysis, this study helps clarify the consistency and durability of treatment effects while also identifying important evidence gaps that should guide future research priorities.

## 5. Conclusions

Overall, the currently available randomized evidence suggests that septoplasty may provide greater improvement in nasal obstruction symptoms and disease-specific quality of life compared with non-surgical management in adults with deviated nasal septum. However, the strength of these conclusions remains limited by the small number of available RCTs, heterogeneity in conservative treatment protocols, and the overall low-to-moderate certainty of evidence. Further large-scale, multicenter randomized studies with standardized outcome reporting and long-term follow-up are needed to better define the comparative effectiveness of septoplasty versus conservative management.

## Figures and Tables

**Table 1:** Characteristics of included studies.

Study ID	Study Design	Blinding Status	Country	Duration of Recruitment	Sample Size, n	Study Arms			Follow-Up	Primary Endpoint
						Surgical	Surgical Definition	Non-Surgical		
Carrie et al. [11]	RCT	Open-label trial; participants and investigators were not blinded	UK	January 2018–December 2019	378	Septoplasty with or without concurrent turbinate surgery	Septoplasty through a closed approach (no external incisions). No grafts allowed. Surgeons could use varied techniques for manipulating the septum. Optional unilateral inferior turbinate reduction is allowed using mucosal-preserving techniques.	Isotonic nasal saline spray and mometasone furoate nasal steroid spray	12 months	Sino-Nasal Outcome Test-22 (SNOT-22) score at 6 months
Srinivasan et al. [12]	RCT	Not blinded; open-label study	India	February 2017–December 2018	140	Septoplasty	Performed under local anesthesia with sedation, Hemi-transfixion incision Elevation of mucoperichondrial flaps Removal of deviated septal cartilage while preserving dorsal and caudal struts Nasal packing used postoperatively.	Topical nasal decongestants and fluticasone nasal spray	6 months	Improvements in VAS, NOSE, SNOT-22, and PNIF scores at 6 months.
van Egmond et al. [13]	RCT	Open-label randomized controlled trial	Netherland	September 2013–December 2016	203	Septoplasty with or without concurrent turbinate surgery	Septoplasty under general anesthesia, Concurrent inferior turbinate surgery was allowed, Surgery performed according to usual local clinical practice (pragmatic design).	local corticosteroids	24 months	Health-related quality of life (HRQoL) measured using NOSE score at 6 months.

**Table 2:** Baseline characteristics of the included participants.

Study ID	Participants		Age (Years)		Male		Baseline NOSE, Mean (SD)		Baseline SNOT-22, Mean (SD)		Baseline PNIF, Mean (SD)		Nasal Obstruction				Septal Deviation				Allergic Rhinitis, n		Asthma, n	
													Unilateral		Bilateral		Unilateral		Bilateral					
	Septoplasty	NS	Septoplasty	NS	Septoplasty	NS	Septoplasty	NS	Septoplasty	NS	Septoplasty	NS	Septoplasty	NS	Septoplasty	NS	Septoplasty	NS	Septoplasty	NS	Septoplasty	NS	Septoplasty	NS
Carrie et al. [11]	188	190	40.3 ± 14.9	39.4 ± 13.9	126	127	70.8 (16.6)	71.7 (16.9)	44.5 (20.8)	44.1 (21.1)	102 (51.2)	102 (49.4)	109 (58%)	113 (59%)	79 (42%)	77 (41%)	150 (80%)	153 (80%)	38 (20%)	37 (19%)	32 (31%)	30 (30%)	24 (24%)	24 (24%)
Srinivasan et al. [12]	70	70	33.75 ± 7.8		73	67	66.6 (22.7)	63.33 (22.7)	18.66 (9.46)	15.66 (9.08)	110 (45.4)	120.83 (47.3)	NA	NA	NA	NA	NA	NA	NA	NA	NA	NA	NA	NA
van Egmond et al. [13]	102	101	39 ± 14	37 ± 15	73	66	67.2 (18.1)	65.5 (19.3)	35.9 (15)	37.9 (16.4)	91.6 (39)	87.1 (40.5)	38 (37%)	40 (40%)	64 (63%)	61 (60%)	77 (75%)	78 (77%)	25 (25%)	23 (23%)	NA	NA	NA	NA

**Table 3:** GRADE outcomes.

Certainty Assessment							№ of Patients		Effect		Certainty	Importance
№ of Studies	Study Design	Risk of Bias	Inconsistency	Indirectness	Imprecision	Other Considerations	Septoplasty	Non-Surgical	Relative (95% CI)	Absolute (95% CI)		
NOSE score. 3 months												
2	randomised trials	not serious	serious ^a^	not serious	serious ^b^	none	165	169	-	MD 7.7 lower (30.01 lower to 14.62 higher)	⨁⨁◯◯ Low	CRITICAL
NOSE score. 6 months												
3	randomised trials	not serious	serious ^c^	not serious	serious ^d^	none	308	312	-	MD 24.8 lower (37.83 lower to 11.78 lower)	⨁⨁◯◯ Low	CRITICAL
NOSE score. 12 months												
2	randomised trials	not serious	not serious ^e^	not serious	serious ^f^	none	199	213	-	MD 17.29 lower (22.29 lower to 12.29 lower)	⨁⨁⨁◯ Moderate	CRITICAL
SONT-22. 3 months												
2	randomised trials	not serious	serious ^g^	not serious	serious ^b^	none	165	169	-	MD 5.6 lower (16.04 lower to 4.84 higher)	⨁⨁◯◯ Low	CRITICAL
SNOT-22. 6 months												
3	randomised trials	not serious	serious ^h^	not serious	serious ^i^	none	315	323	-	MD 11.69 lower (23.2 lower to 0.19 lower)	⨁⨁◯◯ Low	CRITICAL
SNOT-22. 12 months												
2	randomised trials	not serious	not serious ^e^	not serious	serious ^j^	none	213	220	-	MD 9.49 lower (13.14 lower to 5.84 lower)	⨁⨁⨁◯ Moderate	CRITICAL
PNIF. 3 months												
2	randomised trials	not serious	serious ^k^	not serious	extremely serious ^l^	none	165	169	-	MD 1.16 higher (17.61 lower to 19.93 higher)	⨁◯◯◯ Very low	IMPORTANT
PNIF. 6 months												
3	randomised trials	not serious	serious ^m^	not serious	very serious ^n^	none	290	291	-	MD 11.9 higher (9.63 lower to 33.16 higher)	⨁◯◯◯ Very low	IMPORTANT
PNIF. 12 months												
2	randomised trials	not serious	serious ^o^	not serious	very serious ^p^	none	160	167	-	MD 15.42 higher (2.17 lower to 33.01 higher)	⨁◯◯◯ Very low	IMPORTANT

a–p: Downgrading decisions according to the GRADE approach were primarily based on inconsistency due to substan-tial statistical heterogeneity (I^2^ > 50%) and/or imprecision due to wide confidence intervals, small sample sizes, or over-lap of confidence intervals with the line of no effect.

## Data Availability

The analyzed dataset in this research study can be provided by the corresponding author upon reasonable request.

## Supplementary Materials

The following supporting information can be downloaded at: https://www.mdpi.com/article/10.3390/jcm15103930/s1, Figure S1. Trial sequential analysis TSA for primary outcome at 3 months; Figure S2. Trial sequential analysis TSA for primary outcome at 6 months; Figure S3. Trial sequential analysis TSA for primary outcome at 12 months; Table S1. Search Strategy; Table S2. PRISMA CHECKLIST.
